# Well-being of Polish university students after the first year of the coronavirus pandemic: The role of core self-evaluations, social support and fear of COVID-19

**DOI:** 10.1371/journal.pone.0259296

**Published:** 2021-11-16

**Authors:** Elżbieta Turska, Natalia Stępień-Lampa

**Affiliations:** 1 Faculty of Social Sciences, Institute of Psychology, University of Silesia, Katowice, Poland; 2 Faculty of Social Sciences, Institute of Political Science, University of Silesia, Katowice, Poland; Qazvin University of Medical Sciences, ISLAMIC REPUBLIC OF IRAN

## Abstract

**Background:**

The SARS-CoV-2 pandemic represents an unprecedented situation in the most recent history. It has had a number of negative consequences for individuals and for whole societies. Individual effects of the pandemic include not only loss of life and of physical health, but also deteriorated quality of life.

**Objective:**

This study examines the effect of core self-evaluations (CSE), social support and fear of COVID-19 on the well-being of university students in Poland during the COVID-19 pandemic.

**Methods:**

We conducted an online survey on a nationwide group of 1,000 students of Polish universities. The survey was carried out between 1 and 15 March 2021. The respondents filled out the following set of tools: World Health Organization Quality of Life Scale, Core Self-Evaluations Scale, Multidimensional Scale of Perceived Social Support and The Fear of COVID-19 Scale.

**Results:**

The findings showed that core self-evaluations (CSE) were significantly positively associated with well-being in each of the four domains studied: physical health (r = 0.519), psychological (r = 0.763), social relationships (r = 0.465) and environment (r = 0.496). Similarly, social support correlated positively with physical health (r = 0.277), psychological health (r = 0.306), social relationships (r = 0.552) and environment (r = 0.496). Fear of COVID-19 correlated negatively with well-being in the domain of physical health (r = 0.188), in social relationships (r = 0.042) and with regard to the environment (r = 0.071), the correlations were weak. However, the relationship between fear of COVID-19 and well-being in the psychological domain was not confirmed.

**Conclusion:**

The findings point to the significant role of CSE and the role of social support in the perceived quality of life of young people during the pandemic. They provide valuable data concerning individuals who are particularly vulnerable to the adverse psychological effects at the time of the pandemic. They also prove that research conducted to explore other factors protecting individual well-being in difficult situations, including that of the pandemic, should be continued.

## Introduction

The first confirmed case of SARS-CoV-2 in Poland was recorded on 4 March 2020. As a result of the restrictions that were imposed, the majority of Polish university students continued their studies remotely until the end of the 2019/2020 academic year. The summer holiday period brought temporary relief from the difficult pandemic situation. However, in the autumn of 2020, the epidemic situation in Poland deteriorated significantly and, as a result of that, education in the whole 2020/2021 academic year was provided mostly in the form of distance learning. The virus became a real threat in the whole world and many families suffered due to the loss of loved ones who had lost the fight against it. It was also a period, on the one hand, of intense stress and fear of infection, and on the other hand of a sense of powerlessness in the face of the successive restrictions, and of no hope for a rapid change in the situation [1–4]. These feelings were also not alien to students [5], including students of Polish universities. The vast majority of them were confined to their homes and deprived of contact with their educational and social environment, moving their activity nearly entirely to the virtual space. This, in turn, definitely implied a higher risk, compared to the pre-pandemic period, of developing a social media addiction, with all the negative implications of the phenomenon [6].

The impact of COVID-19 on individuals and on the general public was and continues to be profound: from intensive quarantine and social distancing through job loss and financial hardships to loss of health (including psychological health) and life [1,2,4,5,7]. COVID-19 has undoubtedly changed the way in which the contemporary world functions. Brodeur et al. [8] used Google Trends data to analyse terms searched for during the pandemic period. According to the findings, search terms such as boredom, loneliness, worry, and sadness were significantly more frequent, which allowed the researchers to confirm the proposition that the pandemic had a negative impact on well-being and led to a reduction in perceived quality of life. This study focuses on examining evaluations of Polish students’ qualify of life in four core areas (i.e., physical health, psychological, social relationships, and environment domains), one year after the outbreak of the COVID-19 pandemic.

The unprecedented situation caused by the SARS-CoV-2 virus has affected each individual’s daily life and well-being in different ways. It has turned out to be particularly difficult for older people and middle-aged adults [9,10]. However, although younger people are at lower risk of severe COVID-19, and there is a lower risk of COVID-19-related complications in this group [11], they also experience a number of negative consequences of the pandemic. Young people suffer due to social distancing and self-isolation caused by the closure of schools and other dramatic changes in their environment. This has been confirmed by a recent report revealing that the social distancing measures introduced during the COVID-19 pandemic were associated with depression and anxiety in teenagers, and further confirmed by scientific research [12–16].

The aim of this study is to establish the relations between selected individual and social resources: core self-evaluations and perceived social support, and emotional response to threat, operationalised as fear of COVID-19, on the one hand, and the well-being of Polish students on the other hand.

The pandemic is a difficult and stressful situation; in the process of coping with such a situation, researchers emphasise the particular importance of individual resources (traits, characteristics). Individual resources are defined as personal conditions, items, and forms of energy that are either valuable in themselves for survival, or serve as a means to achieve objects of significant value to the individual [17]. Among the psychological resources perceived as important in the process of effective coping in a difficult situation, researchers most often distinguish individual traits and characteristics such as self-efficacy, values, optimism, sense of control, and sense of coherence [18]. In many studies, these traits are indicated as predictors of individual well-being [19,20], and at the same time researchers point out that they are strongly intercorrelated both conceptually and empirically. Taking this fact into account, Judge, Locke, and Durham [21] proposed and identified a single overarching trait, referred to as a higher-order construct, comprising the following individual dispositional variables of the individual: self-esteem, locus of control, self-efficacy, and emotional stability. The construct was named core self-evaluations (CSE).

CSE is conceptualized as the overall, fundamental perception that individuals have about their own worth and capability as human beings [22]. CSE is by definition a higher-order trait, indicated in research for instance as a predictor of job satisfaction [23,24] and life satisfaction [25,26]. The positive association of CSE with life satisfaction (r = 0.54) was confirmed by the results of a meta-analysis by Chang et al. [27]. Kammeyer-Mueller et al. [28], in a meta-analytic review, found that high CSE was associated with fewer perceived stressors and less strain.

The significance of CSE during the COVID-19 pandemic is indicated by the research conducted by Oktaria and Parahyanti in Jakarta on a sample of working and child-rearing individuals. The researchers demonstrated the importance of CSE for the improvement of workplace well-being and as a buffer against the negative impact of work- and family-related burdens [29]. Taking into account the results of previous studies, CSE is expected to be positively related to Polish students’ quality of life evaluations during the pandemic.

External resources, most notably social support, also play an important role in the individual’s adaptation to difficult, crisis situations. Social support influences the perception of stressful events and also neutralises and mitigates their negative effects, helping one to overcome difficulties [30,31]. Most researchers agree that social support has a significant positive impact on individual mental health and well-being [32–36]. Individuals with lower support displayed more symptoms of a bad psychological condition and of depression. They were characterised by a higher degree of anxiety and stress symptoms, as well as by lower sleep quality [37–42]. Based on the above results, we expect that support evaluations will be positively related to the evaluations of the quality of life of Polish students during the pandemic.

Fear is significantly related to the way in which a difficult situation is perceived, to the undertaking of certain behaviours, and to adaptation processes. Its impact on behaviour depends on its severity. Researchers emphasise that high severity of fear disorganises activity and hinders effective overcoming of difficulties, as it favours the perception of many situations as threatening, even if objectively this is not the case, while low severity of fear may have a mobilising effect on the individual’s behaviour [43,44]. Studies have shown that fear levels changed during the pandemic. Initially, they were relatively high, adversely affecting the mental health of individuals [45,46]. In the later phases of the pandemic, especially in the period of declining infection rates, the level of fear decreased [47]. Importantly, the level of fear of COVID-19 in young people compared to older people appears to be relatively lower [48,49]. During the COVID-19 pandemic, fear and anxiety, associated with the threats related to the epidemic situation and its consequences, can be operationalised as the fear of COVID-19 construct [50]. We use this construct in our study, and formulate the expectation that the quality of life of the students surveyed will be negatively related to the level of fear of COVID-19.

The study focuses mainly on the quality of life of Polish students during the coronavirus pandemic. Quality of life is a multidimensional and interdisciplinary concept. Research on quality of life uses both the narrow and the broad approach. In the broad approach, quality of life is satisfaction with life as a whole (well-being), while the narrow approach focuses on satisfaction with selected areas of life. This study used the narrow approach and studied the subjective perception of quality of life in the physical health, psychological, social relationships, and environment domains. Perceived quality of life understood in this way links this concept to psychological well-being, which in this approach is defined as the cognitive and emotional evaluation of one’s own life, including judgements concerning satisfaction, with its individual domains [51].

The following research hypotheses were formulated in relation to the aim of our study:

H1A. CSE will correlate positively with dimensions of quality of life of Polish students in the physical health, psychological, social relationships, and environment domains.
H1B. Perceived social support will correlate positively with dimensions of quality of life of Polish students in the physical health, psychological, social relationships, and environment domains.
H1C Fear of COVID-19 will correlate negatively with dimensions of quality of life of Polish students in the physical health, psychological, social relationships, and environment domains.
H2. CSE, social support and fear of COVID-19 will be significant predictors of quality of life dimensions when their shared variance is statistically controlled.

## Methods

### Participants and procedure

The research was conducted between 1 and 15 March 2021 on a nationwide sample of Polish university students. The data were collected by the professional company BioStat, supporting data collection and specialising in online surveys. Participation in the study was voluntary, and all the participants provided their informed consent to participate. The participants were informed about the possibility of withdrawing from the study without stating the reasons, that their responses would be used exclusively for research purposes, and that the researchers would not have access to their personal data. Participation in the survey was remunerated. Participants were given points which they could subsequently redeem for a cash equivalent.

The current study was reviewed and approved by the Ethics Committee of the University of Silesia in Katowice (permission number: KEUS.95/02.2021).

### Measures

#### World Health Organization Quality of Life Scale (WHOQOL) [52]

The WHOQOL-Bref (World Health Organization Quality of Life Scale (QoL), abridged version), in the Polish adaptation by Jaracz et al. [53], was used to study quality of life in the following four domains: physical domain, psychological domain, social relationships domain, and environmental domain. The version used in the research contains 26 items. Each item is rated on a scale of one to five (1 = very dissatisfied, 5 = very satisfied), with higher scores indicating higher QoL with the exception of three items related to pain and discomfort (item no. 3), need for treatment (item no. 4), and negative feelings (item no. 26). The total score obtained corresponds to individual perceived quality of life in the respective domain. In earlier research, the scale had good psychometric properties. In international research, internal reliability for the individual scales was satisfactory [54–57]. The α values obtained in the Polish research [53] are the following: physical health: 0.81; psychological health: 0.78; social relationships: 0.69; environment: 0.77. In the presented research, α for physical health was 0.72; for psychological health 0.83; for social relationships 0.69; and for the environment: 0.71.

#### Core Self-Evaluations Scale (CSES) by Judge, Erez, Bono, and Thoresen [58]

The current study used the CSES in the Polish adaptation by Walczak and Derbis [59]. The scale consists of 12 statements which the respondents rate using a five-point Likert scale, with 1 = “strongly disagree”, and 5 = “strongly agree”. The final score is the total number of points obtained, with items 2, 4, 6, 8, 10 and 12 that need to be reversed. Cronbach’s α internal consistency coefficient in the study was 0.81. In earlier research, the scale displayed good psychometric properties. Internal reliability in research conducted by the authors of the scale [58] was within the range of 0.81 to 0.87, in Polish research [59] it was satisfactory and ranged from 0.77 to 0.83, while in the presented research α = 0.82.

#### Multidimensional Scale of Perceived Social Support (MSPSS) by Zimet, Dahlem, Zimet, and Farley [60]

The current study used the Polish adaptation of the MSPSS by Buszman and Przybyła-Basista [61]. The scale takes into account the multidimensional nature of perceived social support, taking into consideration its three main sources: significant other, family, and friends. It consists of 12 statements which the respondents rate using a seven-point Likert scale, with 1 = “strongly disagree” and 7 = “strongly agree”. In earlier research, the scale had excellent psychometric properties. The α internal consistency coefficient in the validation study [60] was as follows: overall result– 0.88; significant other– 0.91; family– 0.87; friends– 0.85. In the Polish research, for the overall result its value was 0.89; for the friends subscale– 0.93; for the family subscale– 0.92; for the significant other subscale– 0.87 [61]. In the presented study, the α values were as follows: overall result– 0.91; significant other– 0.92; family– 0.90; friends– 0.93.

#### The Fear of COVID-19 Scale (FCV-19S) by Ahorsu et al. [50]

This scale has been found to be invariant across gender and age groups [62–65]. The research used the Polish adaptation of the FCV-19S by Pilch, Kurasz, and Turska-Kawa [65]. The scale consists of 7 statements which the respondents rate using a 5-point Likert scale, with 1 = “strongly disagree” and 5 = “strongly agree”. Cronbach’s *α* internal consistency coefficient is satisfactory in this study, at *α =* 0.861. In earlier research, the scale had excellent psychometric properties. In the validation study [50] reliability of the tool was satisfactory (α *=* 0.82), in the Polish research [65] the alpha coefficient ranged from 0.89 to 0.85, and in the present study α = 0.86.

### Statistical analyses

The IBM SPSS software (version 26) was used to compute descriptive statistics, internal consistency, correlation and regression analyses. Correlation analysis was performed to verify hypotheses H1A, H1B, and H1C. Cohen’s classification [66] was used to interpret effect size: coefficients of 0.1 represent weak associations, coefficients of 0.3 represent medium associations, and those of 0.5 describe strong associations. A set of multiple regression analyses was performed to establish relationships between predictor variables and each of the well-being domains (i.e., physical health, psychological health, social relationships and environment). The scores on the CSE scale, the FCV-19S, and the overall scores on the MSPSS were chosen as predictors. These variables were related to all the outcomes in the correlational analysis (except the FCV-19S and psychological health). All the models were controlled for demographic variables (age and sex). The assumption for regression analysis were tested before the analyses. The VIF values were < 1.3, and tolerance was < 1, indicating that multicollinearity of the predictors is not a large problem. Also the remaining assumptions for regression analysis were not violated.

## Results

We analyzed data from N = 1000 participants (https://osf.io/p6c3s/, 647 women, 64.7%, M_age_ = 22, 28, SD_age_ = 3.1). The characteristics of the studied group are presented in Table 1.

**Table 1:** Sociodemographic characteristics of the studied group (N = 1,000).

Sociodemographic variables		Number N	Percentage %
Educational/occupational status	Only education	463	46.3
	Education and work	537	53.7
Type of university	Public	832	83.2
	Non-public	168	16.8
Level of studies	First cycle	626	62.6
	Long cycle master’s degree studies	197	19.7
	Supplementary master’s course	177	17.7
Field of studies	Social sciences and humanities (e.g. pedagogy, sociology, management)	464	46.4
	Science and technology (e.g. physics, chemistry, IT)	536	53.6
Current year of studies	1–2	729	72.9
	3–4	251	25.1
	5–6	20	2.0
Population of the locality of permanent residence	Up to 20 thousand	331	33.1
	20,001–50,000	138	13.8
	50,001–100,000	136	13.6
	Over 100,000	395	39.5

The young people studied are not a major risk group for infection with COVID-19. However, 106 people declare that they either had or have a COVID-19 infection, including 78 respondents who were (are) either asymptomatic or mildly ill, and 28 respondents severely and very severely ill. Among those closest to the respondents, as many as 640 people had/have COVID-19. A total of 366 people were mildly ill or asymptomatic, in 250, the disease course was severe or very severe, and 24 died.

Descriptive statistics for study variables and correlations between variables are given in Table 2. The results of the correlation analysis showed that CSE were positively and significantly related to all the well-being domains studied: correlations with the physical health and psychological domains were strong, while correlations with well-being in the domain of social relationships and in the domain of the environment were moderate. These results were congruent with Hypothesis 1A. As predicted in Hypothesis H1B, the relationships between social support and well-being were positive in each of the domains studied: strong for social relationships and moderate for the physical health, psychological and environment domains. These results were congruent with Hypothesis H1B. Fear of COVID-19 correlated negatively with well-being with regard to physical health, with regard to social relationships and with regard to the environment, but the correlations were weak. The relationship between fear of COVID-19 and well-being in the psychological domain was not confirmed. Summarizing, Hypotheses H1A, H1B received full support, while Hypothesis H1C was confirmed partially.

**Table 2:** Descriptive statistics and correlations between study variables.

Variables	*M*	*SD*	1	2	3	4	5	6	7	8	9
1 Physical health	3.79	0.57	-								
2 Psychological health	3.04	0.71	0.582**	-							
3 Social relationship	3.50	0.84	0.410**	0.528**	-						
4 Environment	3.27	0.56	0.595**	0.557**	0.394**	-					
5 CSE	3.02	0.56	0.519**	0.763**	0.465**	0.496**	-				
6 Fear of COVID-19	1.99	0.76	-0.188**	-0.039	-0.042**	-0.071*	-0.053**	-			
7 Support: friends	5.16	1.42	0.277**	0.306**	0.552**	0.302**	0.302**	-0.040	-		
8 Support: family	4.87	1.45	0.393**	0.454**	0.389**	0.438**	0.433**	0.017	0.402**	-	
9 Support: significant other	5.63	1.44	0.228**	0.273**	0.560**	0.213**	0.198**	-0.078*	0.538**	0.379**	-
10 Support: total	5.22	1.14	0.379**	0.436**	0.632**	0.402**	0.394**	-0.042	0.814**	0.753**	0.807**

* p < 0.05

** p < 0.001.

The results of multiple regression analysis are shown in Table 3. The control variables (i.e., age and sex) were significant only for psychological health (older participants and women reported higher psychological health than younger participants and men), but these relationships were weak. CSE and social support predicted significantly and positively all the outcome variables. Fear of COVID-19 predicted (negatively) physical health only. CSE was relatively the strongest predictor of all but one outcomes (i.e., physical health, psychological health, and environment), whereas social support was relatively the strongest predictor of environmental health. The predictors explained together 61% for the variance of psychological health, 46% of the variance of social relationships, 33% of the variance of physical health, and 30% of the variance of environmental health.

**Table 3:** Results of multiple regression analysis.

Predictors	B	lower 95% CI	upper 95% CI	β	t	p
Outcome variable: F1 –**Physical health**						
Age	0.002	-0.008	0.011	0.010	0.387	0.699
Sex	-0.042	-0.104	0.020	-0.035	-1.319	0.187
CSE	0.444	0.387	0.501	0.437	15.221	< 0.001
Support: total	0.098	0.070	0.126	0.196	6.817	< 0.001
Fear of COVID-19	-0.119	-0.157	-0.081	-0.160	-6.128	< 0.001
R^2^ = 0.331; F(5, 994) = 98,348; p < 0.001						
Outcome variable: F2 –**Psychological health**						
Age	0.016	0.007	0.025	0.067	3.388	0.001
Sex	0.066	0.007	0.125	0.045	2.196	0.028
CSE	0.873	0.818	0.927	0.690	31.512	< 0.001
Support: total	0.107	0.081	0.134	0.173	7.880	< 0.001
Fear of COVID-19	0.004	-0.033	0.040	0.004	0.190	0.849
R^2^ = 0.611; F(5, 994) = 311,849; p < 0.001						
Outcome variable: F3 –**Social relationships**						
Age	-0.008	-0.021	0.005	-0.030	-1.257	0.209
Sex	0.016	-0.066	0.099	0.009	0.389	0.697
CSE	0.383	0.307	0.459	0.255	9.851	< 0.001
Support: total	0.392	0.354	0.429	0.531	20.457	< 0.001
Fear of COVID-19	-0.003	-0.053	0.048	-0.002	-0.104	0.917
R^2^ = 0.456; F(5, 994) = 166.416; p < 0.001						
Outcome variable: F4 –**Environment**						
Age	-0.004	-0.014	0.005	-0.023	-0.872	0.383
Sex	0.016	-0.047	0.078	0.013	0.487	0.626
CSE	0.396	0.339	0.454	0.396	13.470	< 0.001
Support: total	0.121	0.092	0.149	0.245	8.332	< 0.001
Fear of COVID-19	-0.027	-0.065	0.012	-0.037	-1.367	0.172

R^2^ = 0.299; F(5, 994) = 84.779; p < 0.001.

## Discussion

The pandemic became and continues to be a difficult and stressful situation for many people, especially those who lost loved ones as a result of COVID-19 infection. This sudden, unexpected and previously unknown situation has disrupted every individual’s perceived quality of life, forcing them to adapt to a number of changes and to new living conditions. At the early stage of the pandemic, the attention of experts around the world focused mainly on explaining how to protect physical health, but as the pandemic spread, the importance of maintaining well-being started to be emphasised increasingly often.

The aim of the study presented in this paper was to determine the relations between CSE, social support and fear of COVID-19 and Polish students’ perceived quality of life during the pandemic. In this study, we use quality of life to define well-being. Quality of life was examined in four domains: physical health, psychological health, social relationships, and the individual’s environment. The findings provide evidence for the importance of individual psychological resources in the form of CSE and perceived social support as factors significantly related to the quality of life of young people in the difficult pandemic situation. This is consistent with the findings obtained by other researchers [25–28].

The results confirm that CSE, as a higher-order factor, plays an important role in protecting the well-being of young people in the current pandemic situation. At the same time, they suggest that particular attention should be paid in this situation to individuals with low CSE levels. Such individuals may be expected to constitute a risk group bearing the highest costs of the pandemic. This is why professional psychological help should be provided precisely to such individuals, and they should be supported in the work aimed at increasing the individual CSE level as a resource significantly protecting their well-being at the time of the pandemic.

In this study, social support correlated positively with all dimensions of well-being and remained their significant predictor in multiple regression analyses. This result is consistent with the findings obtained in other studies, described in the Introduction [34–38]. According to the researchers, a high level of social support may constitute a protective factor against the psychological health problems potentially implied by the SARS-CoV-2 virus pandemic, providing a buffer against negative mental states [39]. Our results provide a further argument for the vital importance of social support in maintaining well-being during the COVID-19 pandemic.

Fear is a natural reaction of the body, pointing to the potential occurrence of danger and mobilising the individual to take actions in order to cope effectively. It often appears in a new, unfamiliar situations, which bring about a number of changes. The COVID-19 pandemic situation has become one of them. We formulated the expectation that the level of fear in a group of Polish university students would be negatively related to well-being. This hypothesis was confirmed only partially. Fear of COVID-19 correlated negatively with physical well-being, although the correlation was weak. In the multiple regression analysis, fear of COVID-19 remained a negative predictor of physical health. Very weak but statistically significant relationships were observed between fear of COVID-19 and well-being with regard to social relationships and environment. However, in the regression analysis, fear of COVID-19 was not a significant predictor of these two dimensions of well-being.

In the present study, fear of COVID-19 was found to be relatively most weakly related to the perceived of quality of life of young people, i.e. Polish university students during the pandemic period which we studied. It can therefore be predicted that it is not a significant risk factor for reduced well-being during the pandemic period in the age group studied. This result is consistent with data presented by other researchers, indicating that the level of fear of COVID-19 in young people is relatively lower compared to older people [47,48]. The generally low level of fear of COVID-19 among young people in the current pandemic situation may be a consequence of the fact that, from the very start of the pandemic, experts have emphasised the high risk of infection among older people and people suffering from chronic diseases. This kind of information can play an important role in reducing fear of COVID-19 in the group of young people, namely those with higher immunity; moreover, young people are generally less fearful of existential threat.

As far as the negative association of fear of COVID-19 with well-being in the physical health domain is concerned, it can be assumed to result for instance from a certain deterioration in health and physical condition experienced by young people forced to temporarily suspend physical exercise or numerous active leisure pastimes they used to engage in previously.

## Limitations

Our study has several limitations. Firstly, an online survey targeted at respondents who are university students weakens the generalisability of its results. The data were collected from the participants on a voluntary basis through an online application. A further limitation may result from the fact that self-evaluation of well-being performed by students may differ from that potentially performed by mental health professionals. Another limitation is the fact that the study used a cross-sectional data collection method which does not legitimise causal conclusions. It is also worth noting the limitation related to the possibility of generalising the research results obtained, due to the fact that certain ways of behaving and experiencing the surrounding reality dependent significantly on the characteristics of that reality. An important dimension of the pandemic situation is represented by the daily reports on the number of infections and deaths. This study was conducted during the so-called third wave of the pandemic in Poland, with the highest number of infections and deaths. It can therefore be assumed that the associations between the studied variables and well-being may depend, to some extent, on the current pandemic situation.

## Conclusions

This study fits in the line of explorations seeking the individual resources and external factors that protect well-being in difficult situations, such as the current pandemic situation. Our study highlighted the important role of individual resources in the form of CSE and social support for the well-being of university students in Poland.

The study provides data, important for both theory and practice, indicating which specific traits and factors are significant for maintaining well-being during social crises, including in particular the current COVID-19 pandemic. The findings from such research may make it possible, among other things, to prepare a specialised support network for people who experience mental health problems. The results we obtained may be relevant for intervention and support programmes to protect psychological health and improve mental resilience. They can also be helpful when making decisions concerning lockdowns and distance learning. On the one hand, social contacts imply spreading the SARS-CoV-2 virus, and on the other hand, as our study showed, they are important and positively influence the well-being of university students in Poland (as well as other social groups, presumably). Therefore, it seems particularly difficult for political decision-makers to choose the right strategy in the fight against the pandemic. In fact, it must be based on finding a balance between, on the one hand, allowing the general public to freely interact, move around, and use social services, and on the other hand, ensuring protection, in particular of individuals at risk of severe COVID-19, against potential infection.
